# Atypical Fibroxanthoma/Pleomorphic Dermal Sarcoma With Osseous Metaplasia: A Series of Three Cases

**DOI:** 10.1111/cup.70046

**Published:** 2026-01-05

**Authors:** Taylor Novice, Yitong Xu, Thomas Brenn, Scott C. Bresler

**Affiliations:** ^1^ Department of Pathology University of Michigan Ann Arbor Michigan USA; ^2^ Department of Pathology and Laboratory Medicine University of Calgary Calgary Alberta Canada; ^3^ Department of Dermatology University of Michigan Ann Arbor Michigan USA; ^4^ Rogel Cancer Center University of Michigan Ann Arbor Michigan USA

## Abstract

Atypical fibroxanthoma (AFX) and pleomorphic dermal sarcoma (PDS) are rare mesenchymal tumors typically arising on sun‐damaged skin of the head and neck in elderly patients. PDS is a more aggressive tumor but with similar demographics, cellular morphology, immunohistochemical features, and genetic findings. The histopathologic diversity and lack of specific immunohistochemical markers for these entities increase the risk of misdiagnosis. To our knowledge, osteoid matrix production has been noted previously in only one case of PDS. We present three additional cases of AFX/PDS with osseous metaplasia, all of which were from the head of elderly patients (91–93 years old). No case recurred or metastasized, thus underscoring the importance of distinguishing this entity from other primary or metastatic tumors with osseous differentiation or metaplasia.

## 1 Introduction

Atypical fibroxanthoma (AFX) and pleomorphic dermal sarcoma (PDS) are rare mesenchymal tumors, typically arising on sun‐damaged skin of the head and neck in elderly patients. Histologically, AFX and PDS feature mitotically active histiocytoid, spindled, epithelioid, and multinucleated giant cells displaying pleomorphic nuclei [1, 2, 3]. While PDS has similar demographics, cellular morphology, and immunohistochemical and molecular features as AFX, it behaves in a more aggressive manner, with extensive involvement of the subcutis, necrosis, lymphovascular invasion, and/or neurotropism [2, 3, 4, 5, 6]. The exact relationship between AFX and PDS remains an ongoing debate; however, given their genetic similarity, they likely represent two ends of a spectrum [3, 4, 5].

AFX and PDS are diagnoses of exclusion, relying on immunohistochemical stains to distinguish from sarcomatoid squamous cell carcinoma, spindle cell melanoma, and leiomyosarcoma [1]. Many histopathologic variants/variations of AFX/PDS have been described, including granular and clear cell types, pigmented lesions with hemosiderin deposition, those with myxoid stroma, and those containing a chondroid matrix, osteoclast‐like giant cells, or prominent sclerosis [1, 3, 7, 8, 9, 10]. To our knowledge, osteoid matrix production was noted in a single dubious case of AFX occurring on the finger of a 47‐year‐old patient and one PDS [11, 12].

The histopathologic diversity and lack of specific immunohistochemical markers increase the risk of misdiagnosis [1]. We present three additional cases of AFX/PDS with osseous metaplasia to underscore this rare finding and avoid misclassification as a primary osseous tumor (Table 1).

**TABLE 1:** Key clinical and histopathologic differences between AFX/PDS with osseous metaplasia and its principal mimics.

Entity	Anatomic site	Age	Infiltrative growth	Osteoid/bone production	Malignant osteoblasts	Mitotic rate	Malignant mononuclear cells	Osteoclast‐like giant cells
AFX/PDS with osseous metaplasia	Head and neck	Elderly	May be present	Present	Absent	Increased	Present	Often present
Primary cutaneous or metastatic osteosarcoma	No site predilection	Wide range	Often present	Present	Present	Increased	Present	Present, not abundant
Giant cell tumor of soft tissue	Superficial or deep soft tissue of the extremities; also trunk, abdomen, pelvis	Adults (20–80 years)	Usually absent	Rarely present, typically at periphery	Absent	Usually low	Usually absent	Present, abundant
Giant cell tumor of bone	Most commonly the epiphyseal region of long bones, particularly near the knee	Young adults (20–40 years)	Usually absent	May be present	Absent	Usually low	Usually absent	Present, abundant

## 2 Case Reports

### 2.1 Case 1

**FIGURE 1:**
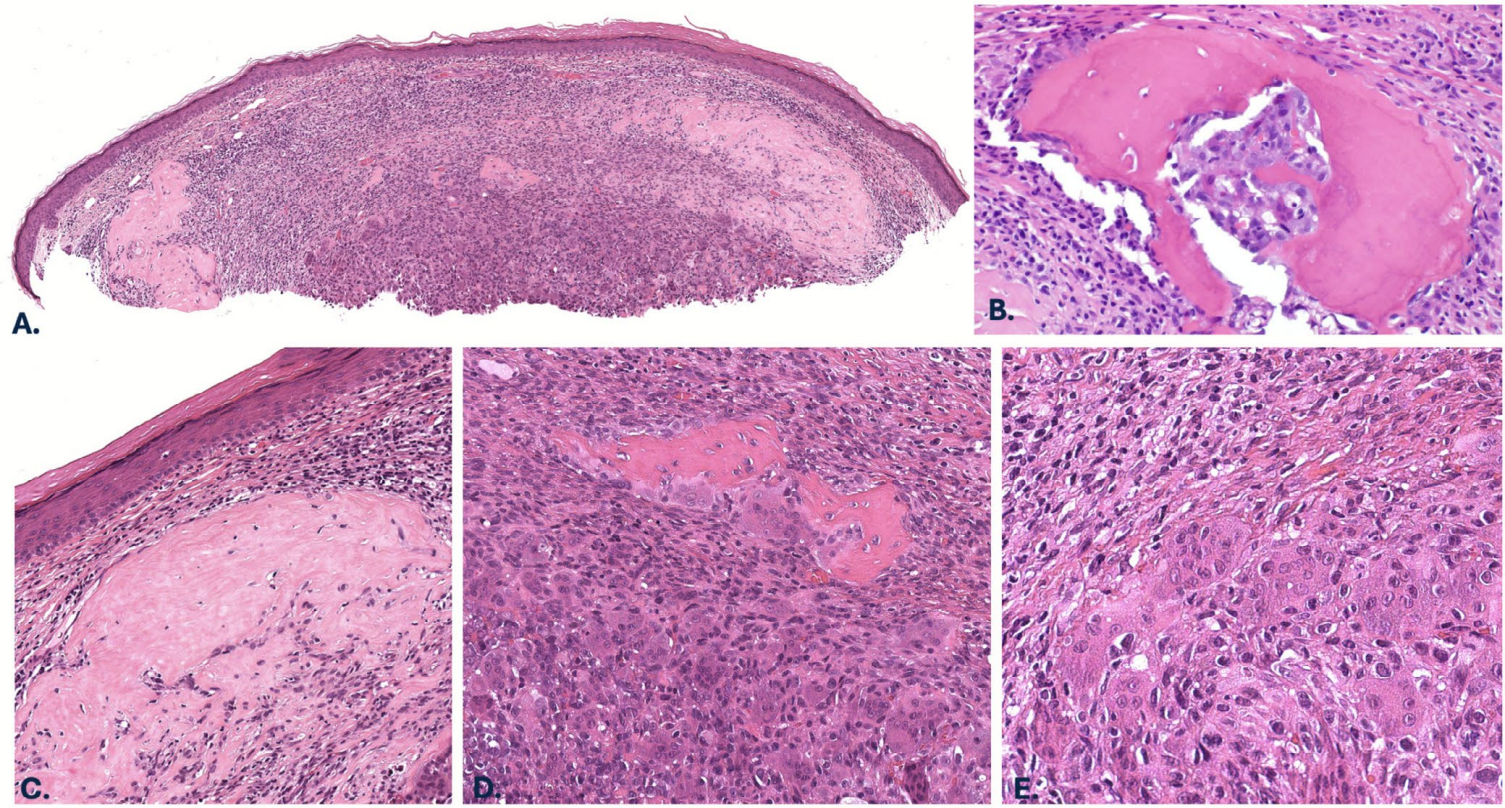
Case 1, histopathologic features. (A) Excisional biopsy specimen of the right cheek nodule demonstrating a nodular intradermal tumor (H&E, 20×). (B) Calcified, mature bone lacking atypical osteocytes (H&E, 400×). (C) Foci of solid eosinophilic material with scattered nuclei consistent with osteoid (H&E, 100×). (D) Mature bone surrounded by mononuclear tumor cells and osteoclast‐like giant cells (H&E, 100×). (E) Within the dermis are atypical and mitotically active spindled to epithelioid cells with large nucleoli and admixed osteoclast‐like giant cells. Occasional mitotic figures are identified (H&E, 200×).

**FIGURE 2:**
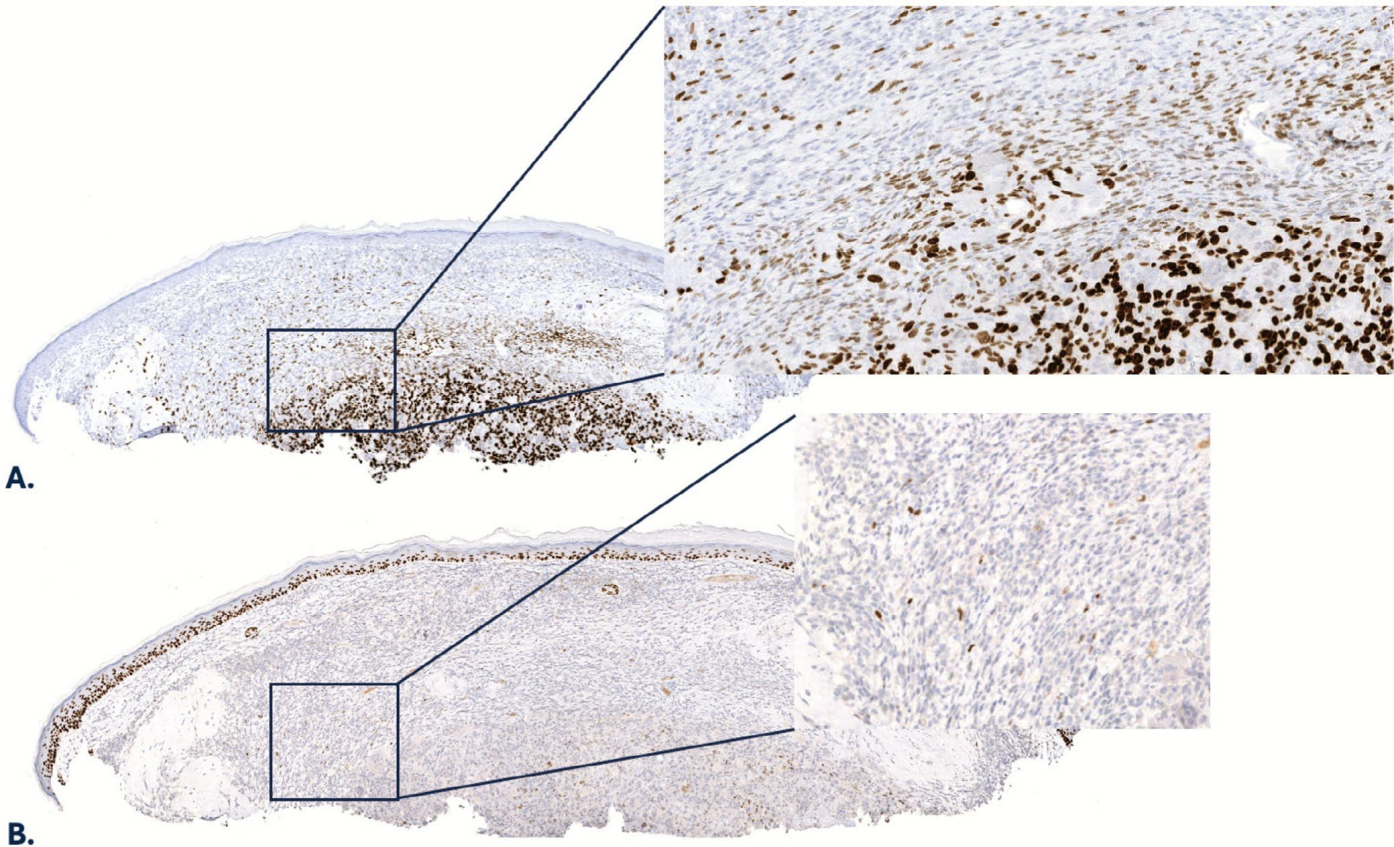
Case 1, immunohistochemical features. Immunohistochemistry showing expression of (A) SATB2 (variable) and (B) scattered p63‐positive cells (20×; inset 80×).

A 92‐year‐old male with a history of a prior AFX of the scalp presented with a pink nodule on the right cheek. A biopsy was performed, which revealed a non‐ulcerated intradermal tumor comprised of haphazardly arranged, mitotically active spindle cells, atypical epithelioid cells with large nucleoli, and osteoclast‐like giant cells. Overlying or adjacent melanoma in situ or squamous atypia were not identified. The tumor extended from just beneath the epidermis into at least the mid dermis but was transected at the base. Accordingly, the presence of subcutaneous involvement could not be assessed. Foci of solid pink material with scattered non‐atypical cells resembling osteocytes, consistent with osteoid, as well as focal mature bone with evidence of calcification were noted (Figure 1). Cytologically bland osteoblasts were seen rimming the foci of bone. Additionally, there was an associated peripheral lymphoplasmacytic inflammatory infiltrate. By immunohistochemistry, tumor cells showed variable intensity of SATB2 expression (Figure 2A) and were negative for pancytokeratin (MNF116), cytokeratin 5/6, S100 protein, and SOX10. PRAME highlighted scattered weakly positive cells (1+ overall, interpreted as negative). A minor subset of tumor cells (< 5%) expressed p63 (Figure 2B). No recurrence was noted 12 months post Mohs micrographic surgery, which was performed at an outside institution, precluding histologic examination of any residual tumor.

### 2.2 Case 2

**FIGURE 3:**
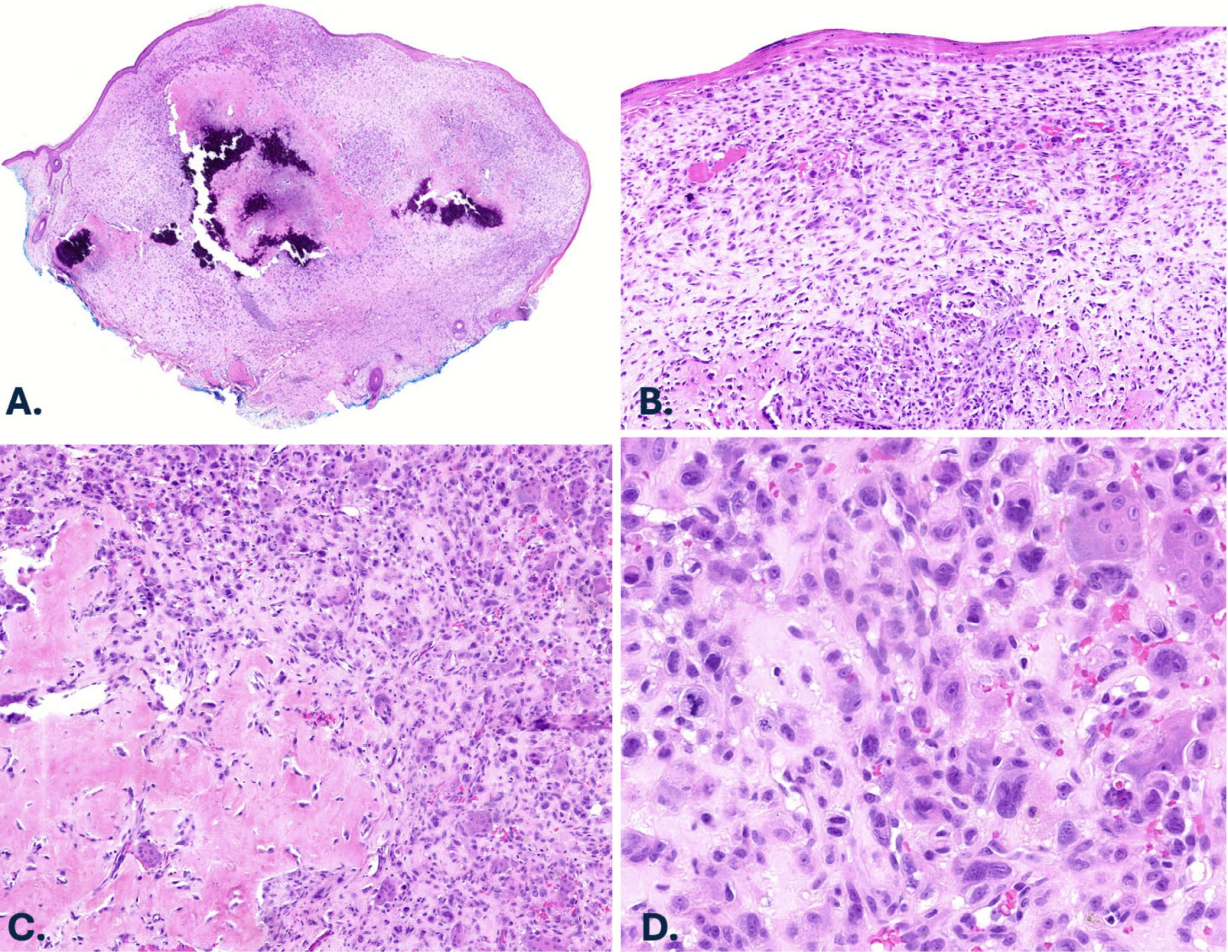
Case 2, histopathologic features. (A) Excisional biopsy specimen of the left ear nodule revealing a poorly differentiated dermal tumor with an extensive associated calcifying osteoid and chondroid matrix (H&E, 20×). (B) Mitotically active dermal pleomorphic spindled and epithelioid cells abutting the epidermis (H&E, 40×). (C) Associated mature bone within the dermis with peripheral benign osteoblasts and surrounded by mononuclear tumor cells and multinucleated giant cells (H&E, 80×). (D) Scattered osteoclast‐like giant cells within the mitotically active mononuclear component (H&E, 200×).

A 91‐year‐old female with a history of melanoma and non‐melanoma skin cancer presented with a left ear nodule. Histopathologic analysis of an excisional biopsy specimen revealed a non‐ulcerated, intradermal tumor without involvement of the subcutis. The tumor extended from just beneath the epidermis into the deep dermis. The neoplasm was composed of markedly atypical spindled and epithelioid cells in a haphazard distribution admixed with osteoclast‐like giant cells (Figure 3). Mitotic figures were frequent and included atypical divisions. An extensive associated osteoid and chondroid matrix with large regions of calcified mature bone was present (Figure 3). Atypical cells were embedded in the osteochondroid matrix; however, benign‐appearing osteoblasts were identified at the periphery of the bone. Although foci of actinic keratosis were seen directly above the tumor, overlying or adjacent melanoma in situ or squamous cell carcinoma in situ were not identified. Tumor cells were strongly positive for SATB2 by immunohistochemistry and were negative for MNF116, CK903/34βE12, p63, S100 protein, and SOX‐10. PRAME showed expression in a small region of the tumor (2+ overall, interpreted as negative). There was no recurrence 60 months post excision.

### 2.3 Case 3

**FIGURE 4:**
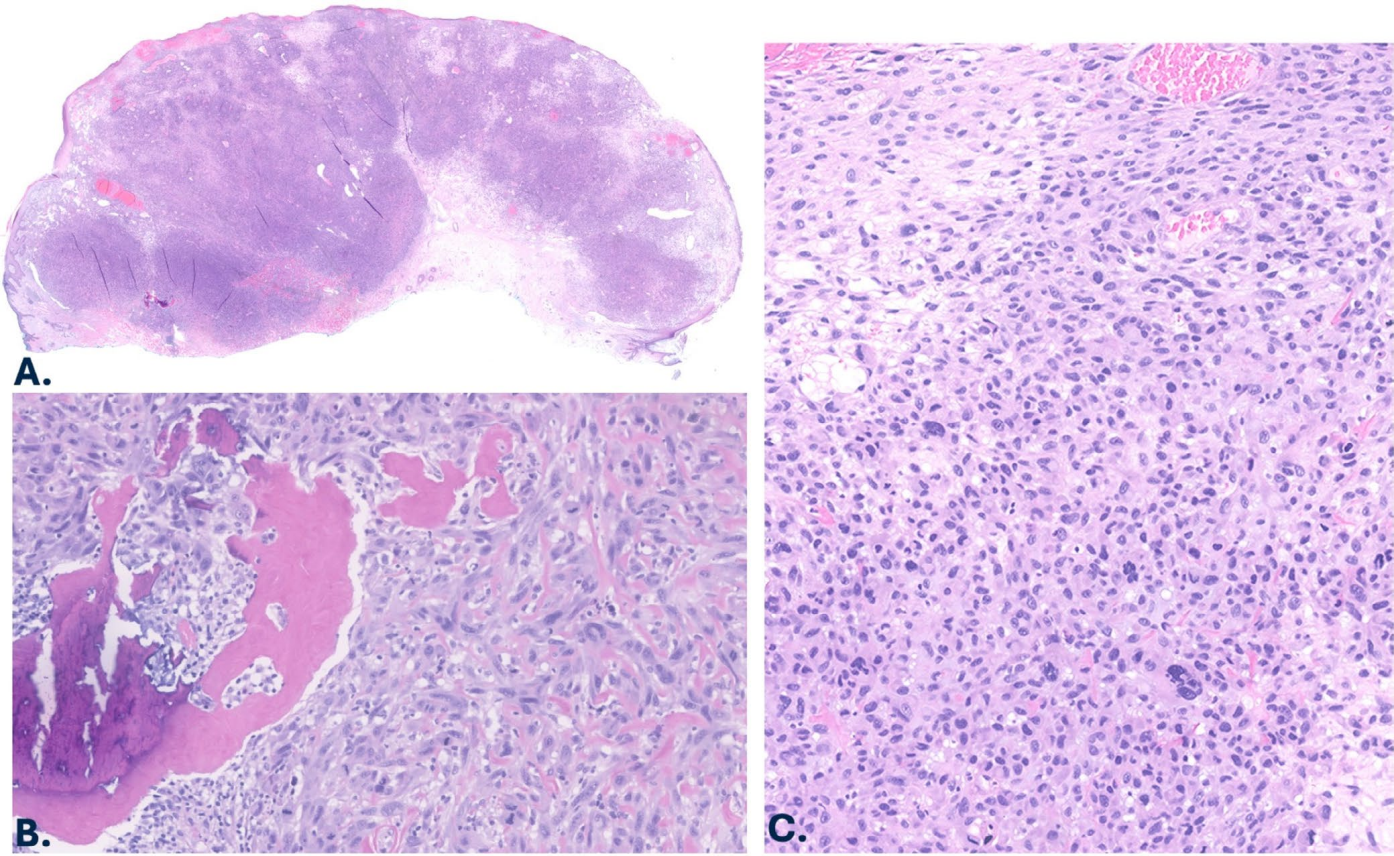
Case 3, histopathologic features. (A) Excision specimen of the right temple nodule showing a poorly differentiated, highly cellular intradermal neoplasm (H&E, 20×). (B) Embedded mature bone surrounded by tumor cells with pleomorphic nuclei and mitotic activity (H&E, 200×). (C) Sheets of mitotically active dermal epithelioid and spindled cells, some containing bizarre and/or multiple nuclei (H&E, 80×).

A 93‐year‐old female presented with a 2.5 × 2.0 cm nodule on the right temple. Histopathologic examination of an excision specimen revealed an ulcerated, poorly differentiated, and densely cellular intradermal tumor extending from just beneath the epidermis to the deep dermis (Figure 4). Mitoses were again numerous and included atypical divisions. Although multifocal actinic changes were seen in the overlying epidermis, associated melanoma in situ or squamous cell carcinoma in situ were not identified. There was an associated osteoid matrix with both woven and focally lamellar bone, the latter lacking atypical osteocytes and with cytologically bland osteoblasts at the periphery. Although some areas of woven bone contained neoplastic cells, most areas did not, supportive of a stromal response rather than true malignant bone. Upon close inspection, the sheets of tumor cells were composed of epithelioid to spindled forms with hyperchromatic, coarse chromatin and palely eosinophilic cytoplasm. Occasional multinucleated tumor cells were present. There were small foci of necrosis noted. Prior partial biopsy had identified lymphovascular invasion, consistent with a diagnosis of PDS. Tumor cells were negative for pancytokeratin (AE1/AE3), CK 5/6, CK7, CK20, Melan‐A, p63, S100 protein, and SOX‐10 by immunohistochemistry. H3K27me3 was retained. There has been no recurrence 36 months post Mohs Micrographic surgery and radiation therapy (35 Gy in 5 fractions).

## 3 Discussion

AFX and PDS are rare dermal tumors with numerous described histologic variants, making them subject to misdiagnosis. A single prior case of an osteoid matrix in a purported (though doubtful) AFX and one in a PDS similar to the findings in our three patients has been reported [11, 12]. Stewart et al. [8] described a case of AFX that had osteoclast‐like giant cells and a small focus of equivocal ossification. In addition, osteoclast‐like giant cells were seen in two of our cases, which have been described previously in at least 16 cases of AFX [1, 7, 8, 9].

In light of the presence of osteoid and mature bone, objectively, the differential diagnosis includes bony tumors such as osteosarcoma, giant cell tumor of soft tissue, and metastatic or contiguous spread of an underlying giant cell tumor of bone (Table 1). The head and neck location and advanced patient age coupled with atypical dermal spindled and epithelioid cells militate against osteosarcoma and giant cell tumor of bone/soft tissue, as does the absence of a prior history of osteosarcoma. Although admittedly challenging to evaluate histopathologically, both primary and metastatic cutaneous osteosarcomas show bone formation by malignant osteoblasts not definitively identified in our cases [13]. Notably, other malignant tumors with osseous metaplasia, such as colorectal carcinoma, may show entrapment of tumor cells within seemingly benign osteoid matrix as observed in cases 2 and 3 of this series [14].

A recent series of 16 cases reinforced the presence of cytologically malignant osteoblasts associated with osteoid matrix in cutaneous osteosarcoma [15]. Interestingly, in all reported cases of primary cutaneous extraskeletal osteosarcoma with clinical follow‐up, the percentage of patients with primary cutaneous osteosarcoma of the head had a high progression‐free survival of 85%, with a 0% local recurrence rate and a 7.7% metastatic rate, faring significantly better than from tumors at other sites. This raises the possibility that at least some of these cases may have in fact represented AFX/PDS with osseous metaplasia. The favorable outcomes in our series of patients further support classification of these tumors on the head and neck of elderly patients as AFX/PDS with osseous metaplasia rather than primary cutaneous extraskeletal osteosarcoma.

The immunohistochemical stains performed in these cases reasonably exclude other common spindle cell neoplasms on chronically sun‐damaged skin and allow for a diagnosis of AFX/PDS [1, 4, 16]. Limitations of immunohistochemistry for the diagnosis of AFX/PDS include examples of AFX being misdiagnosed as angiosarcoma or squamous cell carcinoma due to positivity for CD31 and EMA, respectively [1]. Additionally, strong SATB2 staining in many lesions of AFX can pose a potential pitfall when distinguishing osseous metaplasia in AFX/PDS from osteosarcoma. Indeed, SATB2 expression may be seen in up to 78% of AFX, highlighting its lack of specificity and lack of diagnostic utility in this setting [17]. Negative cytokeratins (such as AE1/AE3, MNF116, and CK903/34βE12), p63, S‐100, and SOX10 immunohistochemical stains help exclude sarcomatoid squamous cell carcinoma and melanoma with osseous metaplasia, both of which can show osteoclast‐like giant cells and SATB2‐positive staining [1, 13, 17]. Rarely, however, AFX/PDS may show p63 expression [3].

AFX/PDS typically occurs in elderly patients, with age ranges from 55 to 95 years old [1, 2, 3]. Our three cases arose on the head of elderly patients, with a narrow age range of 91–93 years old (Table 2). Notably, the previously published case of an AFX with osseous metaplasia on the finger of a 47‐year‐old deviated from the typical AFX/PDS demographic [11]. In a subsequent review of 66 cases of AFX, the forearm was the only case arising at a non‐head/neck location [1]. Furthermore, this case preceded the widespread use of immunohistochemistry. Consequently, an alternative diagnosis is probable given the atypical clinical presentation and lack of an immunohistochemical workup.

**TABLE 2:** Summary of reported cases of AFX/PDS with osseous metaplasia.

Case no.	Sex	Age	Site	Size	Hx of primary osseous tumor	Osteoid matrix	Osteoclast‐like giant cells	Positive stains (IHC)	Negative stains (IHC)	Treatment	Follow‐up	Final diagnosis
1	M	92	Right Cheek	0.5 cm	No	Present	Present	SATB2, subset p63	Pancytokeratin, CK5/6, S100, SOX10, and PRAME	Mohs Micrographic Surgery	No recurrence in 1 year	AFX/PDS with osseous metaplasia^a^
2	F	91	Ear	0.6 cm	No	Present	Present	SATB2	Cytokeratins (MNF116 and CK903), p63, S‐100, SOX10, and PRAME	Re‐excision	No recurrence in 5 years	AFX with osseous metaplasia
3	F	93	Right temple	2.5 cm	No	Present	Not present	None	CD34, pancytokeratin, desmin, epithelial membrane antigen (EMA), ERG, H3K27ME3, myogenin, S100, and SOX10	Mohs Micrographic Surgery followed by radiation therapy	No recurrence in 3 years	PDS with osseous metaplasia
4^b^	M	68	Right side of nose	1.2 cm	N/A	Present	Present	None	Cytokeratins (AE1/AE3 and MNF116), SOX10, S100, desmin, ERGN/A	Re‐excision and adjuvant radiotherapy	No recurrence 3 months post‐surgery	PDS with osseous metaplasia^b^

Abbreviations: AFX, atypical fibroxanthoma; Hx, history; IHC, immunohistochemistry; PDS, pleomorphic dermal sarcoma.

^a^ Subcutaneous involvement cannot be entirely excluded as the excision specimen was not reviewed.

^b^ Previously published case [12].

Given the presence of lymphovascular invasion and focal necrosis in our third case, a diagnosis of PDS with osseous metaplasia was favored. Subcutaneous invasion cannot be excluded in the first case as the debulking excision specimen was not reviewed, typical of Mohs micrographic surgery. To our knowledge, this is the second report of PDS presenting with osseous metaplasia (Table 2) [12].

Overall, AFX is considered a favorable diagnosis, while PDS has a higher propensity for metastasis and recurrence [3, 6, 18]. Although limited by small sample size, the presence of an osteoid matrix did not appear to portend a worse prognosis in our three patients. This underscores the importance of recognizing this osteoid variant of AFX/PDS, which remains a diagnosis of exclusion only to be made after incorporating clinical information and performing necessary immunohistochemical studies.

## Ethics Statement

The authors have nothing to report.

## Data Availability

The data that support the findings of this study are available on request from the corresponding author. The data are not publicly available due to privacy or ethical restrictions.
